# The perinatal microbiota in dogs and cats: a narrative review from human research to veterinary practice

**DOI:** 10.3389/fvets.2026.1817504

**Published:** 2026-04-15

**Authors:** Penelope Banchi, Hanna Mila, Marta Selma-Royo, Smadar Tal, Franck Péron, Virginie Gaillard

**Affiliations:** 1 Faculty of Veterinary Medicine, Ghent University, Merelbeke, Belgium; 2 NeoCare, Université de Toulouse, ENVT, Toulouse, France; 3 Institute of Agrochemistry and Food Technology-Spanish National Research Council (IATA-CSIC), Paterna, Spain; 4 Koret School of Veterinary Medicine, The Hebrew University Veterinary Teaching Hospital, Hebrew University of Jerusalem, Rehovot, Israel; 5 Tel-Hai Academic College, Upper Galilee, Israel; 6 Royal Canin Research Center, Aimargues, France

**Keywords:** bacteria, canine, feline, fetal, microbiome, microbiota, neonatal, perinatal period

## Abstract

Early-life microbial colonization is increasingly recognized as a key determinant of host development and health. While the perinatal microbiota has been extensively studied in humans, comparable knowledge in dogs and cats remains limited. The present review synthesizes current evidence on the perinatal microbiota in dogs and cats, placing it in the context of advances from human microbiome research while emphasizing biological and management factors relevant to veterinary medicine. The prenatal period represents a window of exposure to the maternal microbiota, which is a key contributor to fetal development, while the occurrence of viable microbial colonization of the fetus in healthy pregnancies is not supported by evidence neither in humans nor in companion animals. After reviewing the features of the maternal gut and vaginal microbiota, particular attention is given to the birth process as a major ecological transition, with delivery mode and birth environment shaping early microbial exposure in species-specific ways. The postnatal period is characterized by rapid microbial succession driven by physiological maturation, early nutrition, and environmental factors. We examine the role of colostrum and milk in shaping neonatal gut microbiota assembly, integrating evidence from human studies with emerging data in dogs. We also discuss how maternal care and other environmental exposures contribute to early microbiota development. Finally, we evaluate microbiota-oriented interventions in veterinary settings, including maternal probiotic supplementation, and discuss their potential benefits and limitations based on available evidence. Throughout the review, we discuss current clinical approaches to the perinatal microbiota in companion animals and identify major research gaps. We conclude by emphasizing the need for well-designed longitudinal and ideally multicentric studies integrating maternal, neonatal, and environmental microbiota data and aimed at developing evidence-based microbiome-informed strategies in veterinary practice.

## 1 Introduction

Complex microbial ecosystems influence early development and future health during the perinatal period, which includes the time before, during, and after birth. The *microbiota*, defined as the community of living microorganisms in a given niche, interacts with the host primarily through the production of metabolites and modulation of the immune system. The *microbiome*, by contrast, refers to the collective genetic material of these microorganisms and encompasses not only live microbes but also microbial DNA, cell wall components, and secreted molecules (1). Understanding this distinction is fundamental, as both microbial cells and their molecular products contribute to the maternal signals shaping fetal and neonatal development. During prenatal stages, the maternal gut microbiome may contribute to fetal development and organogenesis via metabolic signals and epigenetic mechanisms (2, 3). At birth and during the neonatal period, the maternal microbiome across various sites (including the gut, vagina, oral cavity, skin, and mammary gland) shapes the initial microbial populations that colonize the newborn. Finally, in the post-natal period, the pioneer gut microbiome of the newborn sets the basis for long-term microbial community structure and host–microbe interactions, according to the *priority effect* concept (4). This suggests that initial microbial colonizers of the neonatal gut influence the trajectory of subsequent microbial community assembly and composition. Specifically, early colonizers can shape the local environment and modulate immune responses, thus affecting which microbes are able to establish later in life. An example of a priority effect is how early arriving bifidobacteria preemptively colonize the infant gut, creating a stable community that influences later microbial development, often preventing other less-adapted bacteria from establishing (5). Bacterial populations play significant roles in the perinatal period and have been recognized as key contributors in the Developmental Origin of Health and Disease (DOHaD) (6). Furthermore, perinatal factors known as modulators of early life colonization are increasingly associated with the incidence of non-communicable diseases (NCDs) in humans, including asthma, allergies, atopy, obesity, neurological, and behavioral disorders (7–9). Similarly, but with far less research available, in companion animals, the perinatal microbiome has been suggested to be associated with neonatal health and could increase susceptibility to disease later in life (10). Indeed, altered early life colonization patterns in dogs have been directly linked to conditions such as fading puppy syndrome and changes in somatic growth rates (11, 12).

These discoveries suggest that interventions targeting the perinatal microbiota hold significant potential for preventative medicine against different conditions in both humans and animals. This moves clinical practices beyond addressing immediate health concerns to a preventive developmental approach targeting the perinatal microbiota and promoting healthy maternal and neonatal gut ecosystems. However, despite growing recognition of the critical role of the perinatal microbiota in future health in humans, research in dogs and cats remains in its early stages. Moreover, although targeted clinical trials in human neonatology demonstrate the feasibility of early-life microbial modulation, equivalent evidence-based protocols in veterinary medicine remain limited, with only a few studies conducted to date. This highlights the need to further develop and adapt such strategies for dogs and cats while accounting for species-specific differences.

The present review aims to (i) synthesize current knowledge regarding the perinatal microbiota in dogs and cats, highlighting comparative insights from human research; (ii) examine the timing and dynamics of pioneer bacterial colonization; (iii) explore the role of maternal health, delivery mode, and early nutrition on neonatal microbiota; and (iv) discuss practical considerations relevant to veterinary settings while identifying critical gaps and future directions for research.

## 2 The prenatal period: maternal microbiome influences fetal development

### 2.1 No evidence of *in utero* fetal microbiota

For decades, the prevailing dogma in both human and veterinary medicine held that the fetal environment was sterile, with microbial colonization beginning only at birth. However, recent advances in microbiome science have challenged this assumption, boosting researchers’ interest in whether microbial exposure might begin in utero and how maternal factors during pregnancy influence offspring health. In 2014, Aagard et al. (13) reported the presence of bacterial DNA in the basal plate of the human placenta prompting research using 16S rRNA sequencing to identify bacterial DNA in fetal proxies, such as the placenta, amniotic fluid, and meconium. Many studies reported the detection of bacterial DNA and proposed that microbial transmission could begin before birth (14–16). However, parallel research denied the presence of bacteria within the same type of samples (17–19) and methodological concerns regarding contamination during sampling and sequencing soon emerged. Fetal samples are considered zero-to-low biomass, which makes them particularly susceptible to contamination during sampling and laboratory processing. Therefore, the detection of bacterial DNA does not necessarily indicate the presence of viable bacterial cells, as further demonstrated by circulating microbial cell-free DNA (cfmDNA) in healthy individuals (20). A consensus has gradually formed in human microbiome research: there is currently no conclusive evidence of viable bacterial colonization of the fetus in utero (21, 22). Similar conclusions have been reached in companion animals, as no viable bacterium has been detected in amniotic fluid samples during healthy gestation (23). However, low amount of bacterial DNA is commonly detected in fetal samples (23, 24) and this may have implications for interpreting positive bacterial qPCR results from amniotic samples in clinical cases. The maternal microbiome affects fetal development and pregnancy outcomes.

### 2.2 The maternal microbiome affects fetal development and pregnancy outcomes

The absence of viable bacteria in the fetal environment does not imply that the microbiome is not relevant to fetal development. On the contrary, maternal microbial communities residing in the gut have systemic influences through both direct and indirect pathways. Microbial metabolites such as short-chain fatty acids (SCFAs), including acetate, propionate, and butyrate, can cross the placental barrier and modulate fetal development via epigenetic mechanisms (25). For example, maternal diets rich in fiber promote SCFA production by the gut microbiota, which has been associated with reduced incidence of childhood asthma and improved neonatal immune programming (26). Accordingly, functional alterations of the maternal gut and vaginal microbiota during pregnancy are increasingly recognized as relevant determinants of pregnancy outcomes and offspring health, although the underlying mechanisms remain incompletely understood (25, 27). Further studies focused on the molecular host-microbe molecular interaction behind associational surveys will be essential to uncover the implications of maternal microbiome for fetal and child development.

In women, the maternal gut microbiota undergoes significant shifts in both composition and diversity during gestation. The gut microbiota shifts from a composition similar to that of healthy non-pregnant individuals in the first trimester to one resembling a disease-associated dysbiosis by the third trimester (28). Furthermore, a large cohort study including more than 2,000 pregnant women reported decreasing alpha diversity and increasing inter-individual variation over gestation (i.e., beta-diversity across different mothers), alongside small fluctuations within vaginal microbial composition and stability within the oral microbiome (29). In general, the vaginal microbiota in women can be categorized into five primary community state types (CSTs) (30), either dominated by *Lactobacillus crispatus* (type I), *Lactobacillus gasseri* (type II), *Lactobacillus iners* (III) or *Lactobacillus jensenii* (type V) or a type IV characterized by high diversity and a mixture of *Gardenella*, *Prevotella*, *Sneathia*, and others (30, 31). Type IV has been associated with disease and adverse pregnancy outcomes (32). While many factors can affect vaginal microbial composition, including hormonal fluctuations, age, ethnicity, sexual behavior, stress, and obesity, the composition of the vaginal microbiota of healthy pregnant women is more stable than that of non-pregnant ones (33, 34), and presents with increased relative abundance of certain species belonging to the genus *Lactobacillus* (32, 35).

The gut microbiota of dogs shares some similarities with that of humans (36), but the vaginal microbiota of companion animals is more diverse compared to that of women (37, 38). In female dogs, the gut microbiota has been reported to be stable during the last third of pregnancy, from day 42 to birth (12–24 h post-partum) by Vilson et al. (39), whereas Garrigues et al. reported a decrease in alpha diversity at day 56 compared to day 28 of gestation (fecal samples collected for three consecutive days at each time point) (40). As regards the vaginal microbiota, recent work reported differences in the frequency of isolates of certain bacterial families (i.e., *Brucellaceae, Aerococcaceae, Enterococcaceae, Micrococcaceae, Aeromonadaceae*, and *Weekesellaceae*) between canine vaginal samples collected 24–48 h before parturition or cesarean section (CS) (*n* = 3) and those obtained between 1 and 6 weeks post-partum (*n* = 80) (41). However, the marked imbalance in sample size between groups limits the reliability of these findings. Taken together, available evidence remains fragmentary, with particularly limited insight into the evolution of the canine vaginal microbiota during gestation and into both gut and vaginal microbiota dynamics in female cats. Future research aimed at modulating vaginal and gut microbiomes during pregnancy, in both humans and companion animals, may therefore provide novel preventive strategies to support fetal development and potentially shape health trajectories in the offspring.

### 2.3 Prenatal interventions affecting the maternal microbiome

Shifts in the gut microbiota during pregnancy can be further triggered by environmental exposures, disease, and various interventions, including dietary modification and the use of “biotics” (e.g., probiotics, antibiotics). Disruption of the gut microbial balance (i.e., dysbiosis) can alter the immune environment at the maternal-fetal interface, thereby contributing to pregnancy complications and affecting offspring development (42–45). Nutrition and gut microbiota are interdependent, with the first shaping microbiota composition and the second participating in food metabolism. Over recent decades, dietary changes and the widespread use of antibiotics have been major drivers of dysbiosis, reducing both microbial diversity and functional capacity (46). This is particularly concerning given that 25% of pregnant women are malnourished (47) and antibiotics represent 80% of treatments prescribed during gestation (48). Conversely, diets rich in fiber, omega-3 fatty acids, and polyphenols are associated with enrichment of butyrate-producing bacterial populations in the maternal gut (49, 50). These metabolites support intestinal barrier integrity, modulate mucosal immunity, and influence systemic immune mediators such as cytokines and immunoglobulins (51). Such dietary patterns have also been linked to differences in the neonatal microbiota, with potential long-term implications for immune development (52, 53). These findings highlight dietary interventions as a powerful strategy to support or restore the gut microbiota during pregnancy. This concept also applies to companion animals, as dietary interventions can modulate the gut microbiota of dogs and cats (54). While nutritional requirements for pregnant dogs and cats are well established (55), there is a lack of studies evaluating how different diets influence the maternal gut microbiota during gestation. Such studies are urgently needed to design dietary strategies that promote a gut microbiome that supports a healthy pregnancy.

Beyond diet, targeted microbiota modulation through probiotic (i.e., living microorganisms with beneficial effects on the microbiota and its homeostasis) supplementation is an emerging strategy to promote maternal and neonatal health (56). However, potential effects of probiotics are strictly dependent on the dosage and strain (57) and the body of literature investigating the effects of their supplementation during pregnancy is characterized by a high degree of methodological and results heterogeneity (58). In a randomized trial involving 30 healthy pregnant women, probiotic supplementation did not significantly alter maternal or infant gut microbial alpha- or beta-diversity, although changes in microbial network structure and functional potential were observed (59, 60). In larger cohorts (*n* = 180 healthy pregnant women), selected probiotic formulations have been associated with reduced maternal and infant infections and shifts toward taxa commonly considered beneficial in the infant gut, particularly among C-section-delivered infants (61). Studies in murine models showed further beneficial effects of gestational probiotics supplementation, including modulation of neurodevelopmental trajectories and promotion of gut development in the offspring (62, 63), although these have not been confirmed in human longitudinal studies.

Prebiotics (i.e., compounds promoting the growth of beneficial microorganisms in the gut) may also be a tool for maternal gut microbiota modulation. Supplementation with galacto-oligosaccharides (GOS) and fructo-oligosaccharides (FOS) from early pregnancy to 6 months postpartum has been shown to alter maternal gut microbiota composition and short-chain fatty acid profiles in women, and to favorably affect the infant gut ecosystem (64). Overall, these findings highlight that probiotic and prebiotic supplementation during pregnancy may be beneficial for both mothers and infants. However, responses appear highly variable and context-dependent, with reports of both beneficial and neutral or adverse microbial outcomes (59, 60, 64).

Research on microbiota-oriented interventions during gestation is emerging in companion animals, as probiotics may represent a promising tool to improve future health in dogs and cats. Recent work has demonstrated that supplementation of pregnant dogs with a yeast-based probiotic containing *Saccharomyces cerevisiae* var. *boulardii* (*S. boulardii*) stabilizes the maternal gut microbiota during the peripartum period by preventing the decline in alpha-diversity (40). Moreover, this intervention promoted the enrichment of SCFA-producing bacteria in the maternal gut on the day following whelping and was associated with lower abundance of potentially pathogenic bacteria. These microbiota changes may underlie the observed increase in fecal secretory IgA levels in supplemented dams, particularly around parturition, reflecting improved mucosal immune homeostasis during this physiologically stressful period. Beyond maternal effects, modulation of the maternal gut microbiota was associated with improved neonatal outcomes. Puppies born to supplemented dams showed a reduced incidence of low birth weight, a known risk factor for neonatal mortality (65), as well as more homogeneous growth trajectories during the late suckling period (days 21–56). In addition, these puppies displayed an attenuated inflammatory profile (lower IL-8: IL-10 ratio) and adequate IgG responses following rabies vaccination (40), suggesting that maternal microbiota modulation during gestation may contribute to shaping early immune development in the offspring.

In contrast, supplementation with lactic acid bacteria has not consistently demonstrated comparable effects. In a study administering *Lactobacillus johnsonii* NCC533 during late gestation and lactation, no significant changes were observed in maternal or neonatal gut microbiota composition (39). These null findings may reflect strain specificity or insufficient dosage but may also be influenced by uncontrolled confounding factors such as diet. Notably, dams in the study by Garrigues et al. received a standardized diet, whereas those in the study by Vilson et al. were maintained on heterogeneous dietary regimens (39, 40).

While further studies would help to clarify the long-term consequences of maternal probiotic and/or prebiotic supplementation for offspring health, in light of promising findings in both humans and dogs, this area of research warrants further development through standardized study designs, while parallel investigations in cats should be initiated, as data are currently lacking.

## 3 Birth is the key moment for pioneer bacterial colonization

### 3.1 Sources of pioneer neonatal gut colonization

Birth is the first significant event for pioneer microbial colonization of neonates in both humans and dogs (11, 19, 23, 66). The maternal microbiome from various sites, such as gut, vagina, skin, nasopharynx, and oral cavity, serves as a source of microbial strains colonizing the newborn (11, 66, 67), although mechanisms of vertical transmission differ between humans and companion animals. While in puppies the pioneer gut microbiota closely resembles that of the dam’s vagina (71.6% match, no significant difference in β-diversity) (66), maternal microbial transmission in humans appears more distributed across body sites. In newborn infants, vaginal bacteria account for approximately 16.3% of the neonatal gut microbiota, whereas maternal gut-derived strains represent about 22.1% (67). Whole-genome sequencing (WGS) metagenomic profiling further revealed that gut-derived strains persist longer and are ecologically better adapted to the infant gut than microbes originating from other sources (67). In contrast, vaginal bacteria exhibit much shorter persistence, declining rapidly within the first week of life (67–69). The biological reasons underlying this species-specific difference in maternal microbial transmission remain unclear.

The importance of the dam’s vaginal microbiota in shaping pioneer gut colonization in puppies has been recently emphasized by a study linking vaginal dysbiosis at natural birth to impaired neonatal outcomes (70). Garrigues et al. identified three distinct vaginal microbiome profiles (clusters 1–3) differing in alpha- and beta-diversity in female dogs at day 1 post-partum. A diverse vaginal microbial profile enriched in *Moraxellaceae* (particularly *Psychrobacter* spp.; cluster 1) was associated with better neonatal outcomes, whereas reduced alpha-diversity with dominance of *Enterobacteriaceae* (particularly *Escherichia coli*; cluster 3) correlated with adverse outcomes, including higher stillbirth and neonatal mortality rates (18% in cluster 3 versus 4% in cluster 1 for both parameters) and slower early growth (twofold lower weight gain in cluster 3 compared to cluster 1 during the first 2 days of life) (70). These differences in the perinatal vaginal microbiome could thus contribute to infections or inflammatory responses in the highly vulnerable neonate, leading to severe conditions like fading puppy syndrome. This understanding presents a critical actionable opportunity for veterinary practice. It implies that pre-breeding or early gestational screening of the vaginal microbiome in dogs could serve as a powerful diagnostic tool to identify at-risk dams, provided that further research is conducted. Data in queens are currently lacking, and dedicated studies in this species are warranted.

### 3.2 Impact of delivery mode and environment on vertical microbiota transmission

Various maternal and environmental factors influence gut colonization dynamics in the first hours after birth, including gestational age, genetics, place of birth (e.g., hospital or home), antimicrobial exposure, and delivery mode (71–73). Among these, delivery mode plays a major role in shaping the initial microbiota in humans (72). Vaginal delivery enhances mother-infant microbiota vertical transmission (74), exposing neonates to maternal gut and vaginal microbes and resulting in greater gut microbial abundance and diversity immediately after birth compared with cesarean section (CS) (75). CS-born neonates showed a delayed colonization of the *Bacteroides* genus (76) and are more likely to harbor opportunistic pathogens during the neonatal period (CS 83.7% vs. 49.4% after vaginal delivery) (72), with profiles enriched in *Enterococcus*, *Enterobacter*, and *Klebsiella* (71, 77). Interestingly, no differences in initial microbial seeding have been observed between CS performed with or without labor onset (78). These temporal shifts create priority effects that modulate microbial succession and gut epithelial development in distinct ways between CS-born and vaginally delivered infants (79). Overall, the influence of the delivery mode on gut microbiota of children persists throughout the neonatal period (77) and has been associated with long-term health outcomes. Specifically, children born via CS show an increased risk of asthma, childhood overweight, and obesity, all suggested to be associated with altered microbial colonization (80–82).

Delivery mode may be an important factor affecting microbiota acquisition in companion animals as well. Studies in cats are currently lacking, whereas research in dogs are limited. Immediately after birth, meconium samples from vaginally delivered puppies yielded bacterial growth in 90% of cases, compared to only 37% of samples obtained after elective CS (24, 66). Furthermore, while the microbiota of vaginally delivered puppies closely resembled the dam’s vaginal microbiota, that of CS-born puppies showed partial similarity with both the dam’s vaginal and oral mucosa (49.9–55.5%, including emergency and elective CSs) (11, 66). Interestingly, some mild differences were found in the prevalence of bacteria belonging to the family *Enterobacteriaceae* and to the genus *Staphylococcus* at different post-natal time-points based on the delivery mode in puppies (83). Nevertheless, further longitudinal studies using molecular methods (e.g., 16S sequencing, shotgun metagenomics) are needed to clarify the impact of delivery mode on the neonatal gut microbiota of companion animals. In addition, the role of place of birth in microbiota acquisition and development should be addressed, as this represents an affecting factor in humans (50, 71). With some country-specific exceptions, fewer than 1% of babies in Europe are born at home (84), whereas companion animals are typically born either in a domestic environment or breeding kennels. Birth in veterinary clinics is limited to cases of dystocia requiring obstetrical assistance or CSs. The contrasting conditions of CS (high use of antibiotics and disinfectants, hospital-associated microbiota), kennel births (frequent disinfectant use and high animal density), and home births (low animal density and lower disinfectant use) are therefore likely to contribute significantly to differences in microbiota acquisition in puppies and kittens. In dogs, the extreme variability in body size across breeds represents an additional species-specific factor that may influence early microbial colonization and warrants further investigation.

### 3.3 Emerging methods to restore vertical transmission

Fecal microbiota transplantation (FMT) has recently emerged as a promising strategy to restore vertical microbiota transmission in cesarean-delivered infants. In a proof-of-concept pilot study including seven mother-infant pairs, maternal FMT was administered by inoculating the first breast milk with a diluted fecal suspension containing 10^6^–10^7^ viable cells (85). This approach exploited breastfeeding as a natural vehicle for mother-to-infant microbial transfer and successfully corrected the disrupted colonization typically observed after CS delivery. Specifically, FMT restored the early acquisition of *Bacteroides* spp. and normalized the delayed expansion of *Bifidobacterium*, while mitigating the enrichment of opportunistic taxa such as *Enterococcus*, *Enterobacter*, and *Klebsiella* observed in CS-born children (77, 85). Besides transiently elevated C reactive protein (CRP), no complication was reported in the short-term. However, this procedure is still controversial due to the risk of pathogen transmission coupled with the immature neonatal immune system. Before adopting this procedure in clinical practice, larger studies are needed to validate safety, define optimal dosing, and assess whether early-life FMT reduces later-life disease risk. FMT has been associated with faster resolution of diarrhea and shorter hospitalization times in older puppies (between 3 and 5 months of age) diagnosed with canine parvovirus infection (86), however no study has investigated FMT as an intervention for neonatal microbiome restoration in dogs and cats.

Vaginal seeding is another controversial strategy to restore vertical microbial transmission in infants delivered by CS. The practice consists of collecting maternal vaginal fluids on sterile gauze prior to surgery and subsequently wiping the newborn’s face, mouth, and body to mimic the microbial exposure of vaginal birth. Observational human studies have reported partial restoration of neonatal microbial profiles following vaginal seeding, with skin and oral microbiota enriched in vaginal taxa such as *Lactobacillus* and *Bacteroides* (87). However, *Lactobacillus* and *Bacteroides* in the gut microbiota were slightly yet not significantly elevated at birth and 6 months (88). At present, the efficacy and safety of this intervention remain unproven in humans, as no large randomized controlled trials are available (89). Safety concerns are considerable, since maternal pathogens (e.g., Group B *Streptococcus*, HIV, herpesviruses) may be transmitted even when asymptomatic, and at least one case report has linked neonatal Herpes Simplex Virus infection to unsupervised seeding (90). For these reasons, vaginal seeding should not be practiced until its risks and benefits are clarified by further research. Given the prominent role of the dam’s vaginal microbiota in shaping pioneer gut colonization in dogs, vaginal seeding could be hypothesized as a potentially more effective intervention for microbiota restoration in CS-delivered puppies than in humans. However, a recent experimental study in dogs applied vaginal seeding, transferring vaginal secretions from dams to the skin and mouth of CS-delivered puppies using sterile gauze and reported no differences in bacterial isolation in culture between treated and untreated puppies (83). Further investigations applying molecular techniques are needed to comprehensively assess microbial dynamics before definitively excluding the potential value of this intervention in dogs and cats. Moreover, future veterinary clinical trials evaluating vaginal seeding should incorporate rigorous maternal pathogen screening and standardized donor and application protocols before any consideration of clinical translation as proposed in human medicine.

## 4 Evolution of the postnatal microbiota

The postnatal period represents a phase of rapid ecological succession. Immediately after birth, the neonatal intestine undergoes intense changes to adapt from fetal to extrauterine life, including an increase in circulation, motility, and nutrient digestion (91). These changes reshape the intestinal habitat and the selective pressures acting on the gut microbiota. In humans, during the first days of life, the neonatal gut is typically dominated by facultatively anaerobic bacteria, such as members of the *Enterobacteriaceae*. These early colonizers are progressively replaced by strictly anaerobic taxa, particularly *Bifidobacterium* species, associated with beneficial effects such as inhibition of pathogens growth, immune stimulation, and modulation of bacterial genes responsible for antimicrobial resistance (92, 93). This microbial succession is thought to be driven by the consumption of oxygen by enterocytes and aerobic bacteria, combined with intestinal barrier closure and epithelial maturation, leading to conditions that favor obligate anaerobes (92).

Beyond physiological drivers, early nutrition plays a key role in postnatal microbiota development. In humans, microbial species detected in breast milk account for a substantial proportion of the infant gut microbiota early in life, representing over 40% of gut species richness during the first week, with this contribution progressively declining over the first year as the infant gut microbiota diversifies (50). Furthermore, colostrum and milk do not only supply bacteria but also oligosaccharides, vitamins, hormones, and immunoglobulins that support microbial colonization and immune maturation (94). These bioactive components are believed to drive divergent colonization trajectories in breastfed neonates and formula-fed ones. Breastfed infants typically show a dominance of beneficial commensal bacteria, particularly *Bifidobacterium* and *Lactobacillus*, whereas formula-fed infants more frequently harbor potentially pathogenic taxa such as *Clostridioides* and members of the *Enterobacteriaceae* (95). Breastfeeding is also associated with lower antimicrobial resistance genes compared to formula feeding (93).

Maternal contributions to infant microbiota development extend beyond breast milk, as microbial communities from multiple maternal body sites, including the skin and nasopharynx, have been shown to shape early-life microbial colonization (74, 96). Alongside maternal influences, the father represents a stable source of microbial strains for the infant gut, with a contribution comparable to the maternal one by one year of age (97). Finally, other factors related to household composition are associated with neonatal microbiota composition in humans (98). Among these, exposure to household pets has been shown to increase neonatal gut microbial richness and diversity and to promote the enrichment of taxa such as *Oscillospira* and *Ruminococcus*, which have been negatively associated with childhood atopy and obesity (99). Although further research is needed to clarify mechanisms and long-term outcomes, these findings suggest that the presence of companion animals in the household may positively influence infant health by contributing to early-life gut microbiota development.

The development of the gut microbiota in puppies and kittens is a dynamic process as in humans, and it is shaped by similar factors such as maternal influences, birthweight, nutrition, and the environment (100, 101). Longitudinal studies show that the neonatal gut microbiota evolves rapidly in the first weeks of life (102, 103), with a marked increase in *Peptostreptococcaceae*, *Pasteurellaceae*, and *Clostridiaceae* occurring by 8 h of life, reflecting the transition from an aerobic to an anaerobic environment, similarly to what occurs in human neonates (104). Overall, from birth to weaning, the gut microbiota of puppies evolves from a community dominated by aerobes and facultative anaerobes to one enriched in obligate anaerobes, with an overall increase in microbial diversity from 2 to 56 days of life and a decreased resemblance to the microbiota of the dam (102, 103). Nevertheless, the dam remains a key influence for early microbiota development, and her colostrum and milk contribute to gut development through bioactive components that are similar to that of humans (105). Analyses of colostrum and milk samples by 16S rRNA sequencing revealed that the two present similar composition and consistently low levels of *Bifidobacterium* in dogs (106), while culture-based studies have identified shared bacterial isolates between colostrum and meconium samples. Such overlapping isolates were not only detected after colostrum ingestion, but also in meconium collected prior to any maternal care (103, 107). Differently from humans, culture-independent sequencing methods revealed that the canine colostrum microbiota has greater similarity to the dam’s vaginal microbiota than to her gut microbiota (70). Overall, these findings suggest that similar bacteria may be encountered by newborns during vaginal delivery and early feeding. Therefore, the use of milk replacers may alter neonatal gut microbiota composition in puppies, as recently reported in kittens (108).

In addition to feeding, the dam likely contributes to the offspring microbiota through other pathways. Maternal licking during nursing transfers microbial populations from the dam’s oral cavity, while close physical contact between the neonate and the dam introduces skin-associated microbes. Overall, this key role of the dam is supported by evidence in dogs showing that littermates share similar gut microbiota with each other and with their mother in early life. However, this similarity progressively decreases with age, approaching weaning (103) when dietary changes become the dominant driver of microbial composition.

While research on neonatal microbiota in companion animals is gradually emerging following human footsteps, important differences between human neonates and puppies and kittens should be considered. Companion animals are typically born in litters, resulting in intense sibling-sibling contact, whereas humans are usually singletons. In addition, the interval from birth to weaning is markedly shorter in dogs and cats (starting at 3–4 weeks) compared with humans, who rely exclusively on milk for the first fourth-six months of life, leading to different temporal windows for dietary-driven microbiota maturation. Postnatal paternal influence on microbiota assembly may also differ, as contact between sires and offspring is often limited in companion animals, whereas continuous father-infant contact is common in humans. Finally, differences in environmental exposure (e.g., indoor versus kennel or household settings) may further contribute to species-specific trajectories of neonatal microbiota development.

## 5 Practical approach to the perinatal microbiota in veterinary settings

While research is undoubtedly growing in companion animals, the clinical approach to the perinatal microbiota should be cautious, encompassing careful dam selection, strategic dietary management, detection of at-risk neonates, judicious use of antimicrobials, thoughtful delivery management, and supportive neonatal care.

### 5.1 Pre-breeding screening and dam health

A cautious approach is advised when selecting dams for breeding. It is prudent to avoid dams with a history of inadequate maternal behavior, chronic infections such as vaginitis or enteritis, or those at a high risk of dystocia. These factors can negatively impact the dam’s overall health and microbiota, indirectly affecting the offspring’s microbial seeding and early development.

### 5.2 Dietary strategies and supplementation during pregnancy

Sudden dietary changes during gestation may cause imbalances in the dam’s gut microbiota and should therefore be avoided. High-quality, consistent nutrition, in line with FEDIAF or AAFCO recommendations, is advised, and some dietary regimens, such as BARF (Bones and Raw Food) diets, should be considered with caution in pregnant animals. While these diets may increase palatability, which can be advantageous in late pregnancy, their benefits remain poorly documented (109). Dogs fed raw diets develop distinct microbiome and metabolome profiles (110, 111), yet these changes have not been conclusively linked to improved health outcomes. In pregnant dams, such microbiota shifts may indirectly affect fetal development and the pool of microorganisms that is transmitted to the offspring at birth. Raw diets are also associated with increase carriage of *Enterobacteriaceae* and other potentially pathogenic or antimicrobial-resistant bacteria in the gut microbiota (109), which may be vertically transmitted to neonates during parturition or early maternal contact. Given the vulnerability of the neonatal period and the importance of controlled maternal microbial ecosystems for optimal early-life colonization, current evidence does not support the use of BARF diets during pregnancy in dogs and cats.

Beyond balanced diets, the use of “biotics” (prebiotics and probiotics) offers a promising strategy for optimizing maternal and neonatal microbiota. Evidence from humans shows that maternal dietary interventions and probiotics supplementation can influence milk microbiota and composition, with potentially positive effects on infant health (112–114). In dogs, daily supplementation of pregnant females with a pre- and probiotic combination (tablets containing *Enterococcus faecium* 3.46 × 10^8^ CFU, *Lactobacillus acidophilus* 1.03 × 10^10^ CFU, mannan-oligosaccharides MOS 48.6 mg, fructo-oligosaccharides FOS 480 mg; one tablet per 10 kg of body weight) significantly improved the concentration of IgG, IgM, and IgA, with best results when supplementation occurred for the last 4 weeks of gestation compared to shorter duration and to the control group (115). Daily administration of the yeast *Saccharomyces cerevisiae* var. *boulardii* during the second half of gestation have a positive effect on the maternal microbiota profile (higher abundance of SCFAs-producing bacteria) that most probably explains the improved colostrum and milk gross energy observed in the same study (respectively 1.2and 1.4 ME/g in yeast-supplemented females vs. 1.4 and 1.6 ME/g in placebo group) (40). Moreover, a positive effect of maternal supplementation with yeast was observed in puppies presenting a more benefic gut microbiota profile (higher abundance of lactic bacteria between 2 and 56 days of age compared with placebo group) and more homogenous growth rate. While positive results observed with *S. boulardii* (40, 104) and lactic acid bacteria strains (115) demonstrate that evidence-based, strain-specific interventions can be effective in dogs, the current body of evidence in companion animals remains limited and heterogeneous. Although some studies, including longitudinal designs with adequate sample sizes, provide valuable insights, others rely on small cohorts and less robust methodologies. Considerable variability in probiotic strains, dosages, timing of administration, and sampling protocols limits cross-study comparability. Furthermore, no studies are currently available in pregnant queens, and it is important to recognize that not all probiotics are equivalent in dogs. Products lacking demonstrated efficacy in pregnant animals, as well as formulations designed for humans, should therefore be avoided. Instead, evidence-based, strain-specific approaches are warranted.

### 5.3 Avoidance of unnecessary antimicrobials during gestation/lactation

In line with current regulations (e.g., Reg. EU 2019/6) and in the One Health antimicrobial resistance framework, the preventive use of antibiotics before and during gestation should be avoided. Antimicrobial treatment of breeding female dogs, even when based on vaginal swab culture results, in the absence of clinical signs, is discouraged. The vagina has its own microbiota that can vary according to the age, body size, and living environment (38, 116, 117). Antimicrobials can significantly disrupt the vaginal and gut microbiota of the dam, with unknown but potentially detrimental consequences for both the mother and the offspring. Furthermore, their use contributes to shaping the resistome (i.e., the collection of antibiotic resistance genes within the microbiome). Evidence from human medicine shows that antibiotic administration during CS can increase the prevalence of antibiotic resistance in early life (93), raising concern that similar effects may occur in companion animals.

### 5.4 Delivery management and seeding

Promoting natural whelping should be prioritized whenever medically feasible, as vaginal birth ensures optimal vertical transmission of maternal microbiota to the neonate. Although the gut microbiota of newborns largely resembles the dams’ vaginal microbiota, providing a theoretical basis for vaginal seeding in c-section delivered puppies, the safety and efficacy of this approach remain unproven. Accordingly, vaginal seeding should not be used outside controlled research settings given the risk of transmitting pathogens to immunologically vulnerable neonates. FMT has shown promising results in older puppies with parvovirus (86), but its use in neonatal dogs and cats has not been tested.

### 5.5 Colostrum management

Prioritizing colostrum intake is crucial for proper gut microbiota shaping in newborns. Colostrum should ideally be administered within the first 8 h after birth, and controlled intake must be ensured within the first 24 h (118). The adequacy of colostrum intake can be evaluated by monitoring growth, aiming for more than 0.5% increase per suckling session and a growth rate of 0–2 days greater than 4%. Colostrum serves as a source of bacteria, stimulates early immunity, and contains vital components such as milk oligosaccharides that promote bacterial growth, and growth hormones that accelerate intestinal maturation and facilitate bacterial adhesion. Furthermore, immunoglobulins and other immune factors present in colostrum selectively enhance the growth of specific beneficial bacteria while limiting the proliferation of others.

### 5.6 Detection of conditions associated with altered gut microbiota

Early detection of at-risk puppies and kittens is a key strategy to reduce neonatal mortality rates in breeding kennels. In puppies, some conditions associated with increased risk of mortality, such as low birth weight (LBW) and fading puppy syndrome, have also been linked to alterations in early gut microbiota composition. LBW puppies have been reported to show neonatal mortality rates that are more than twice those of normal birth weight ones (65) and are more frequently colonized by facultative anaerobes such as *Escherichia coli* and *Clostridium perfringens*, species associated with increased susceptibility to gastrointestinal disorders and increased neonatal mortality (119). In contrast, high birth weight (HBW) puppies exhibit earlier maturation of their gut microbiota, characterized by a greater abundance of taxa such as *Faecalibacterium* and *Bacteroides,* that have been proposed to be beneficial for the host health (119). Therefore, birth weight is a key tool to detect at risk neonates and could potentially serve as biomarker of an altered early gut microbiota requiring support.

The relationship between altered gut microbiota and mortality risk is further supported by data showing that puppies affected by fading puppy syndrome display a distinct microbial profile, including an increased Proteobacteria/Firmicutes ratio and higher relative abundance of *Pasteurellaceae*, alongside a reduction in *Clostridia* and *Enterococcus* compared to healthy ones (12).

Further research is needed to disentangle whether early microbial alterations are a cause or a consequence of adverse neonatal outcomes, and to determine whether early microbiota profiling could be implemented as a prognostic tool in veterinary practice, provided that fast, portable sequencing platforms become available. Moreover, in this framework, microbiome-targeted interventions may represent a promising supportive strategy. Available evidence suggests that maternal yeast supplementation during gestation may support a balanced maternal and neonatal microbiota in dogs, with potential benefits for early immune development and growth. These findings highlight the potential strain-specific maternal probiotic interventions as microbiota-oriented strategies to improve neonatal survival and early life health, while encouraging further targeted studies.

Finally, specific situations such as the use of milk replacers or the management of orphaned neonates may significantly alter early microbial colonization, as reported in kittens fed with milk replacers (108). Unlike maternal milk, milk replacers lack both live microbes and bioactive compounds such as oligosaccharides and immunomodulatory factors, which are known to shape gut microbiota development (94). Similarly, orphaned neonates are deprived of maternal microbial transfer through grooming, close contact, and the dam’s environment. These conditions may result in altered microbial colonization and evolution. However, evidence in dogs and cats remains limited, highlighting the need for targeted studies and microbiota-oriented management strategies in these situations.

## 6 Conclusions and future directions

**Figure 1:**
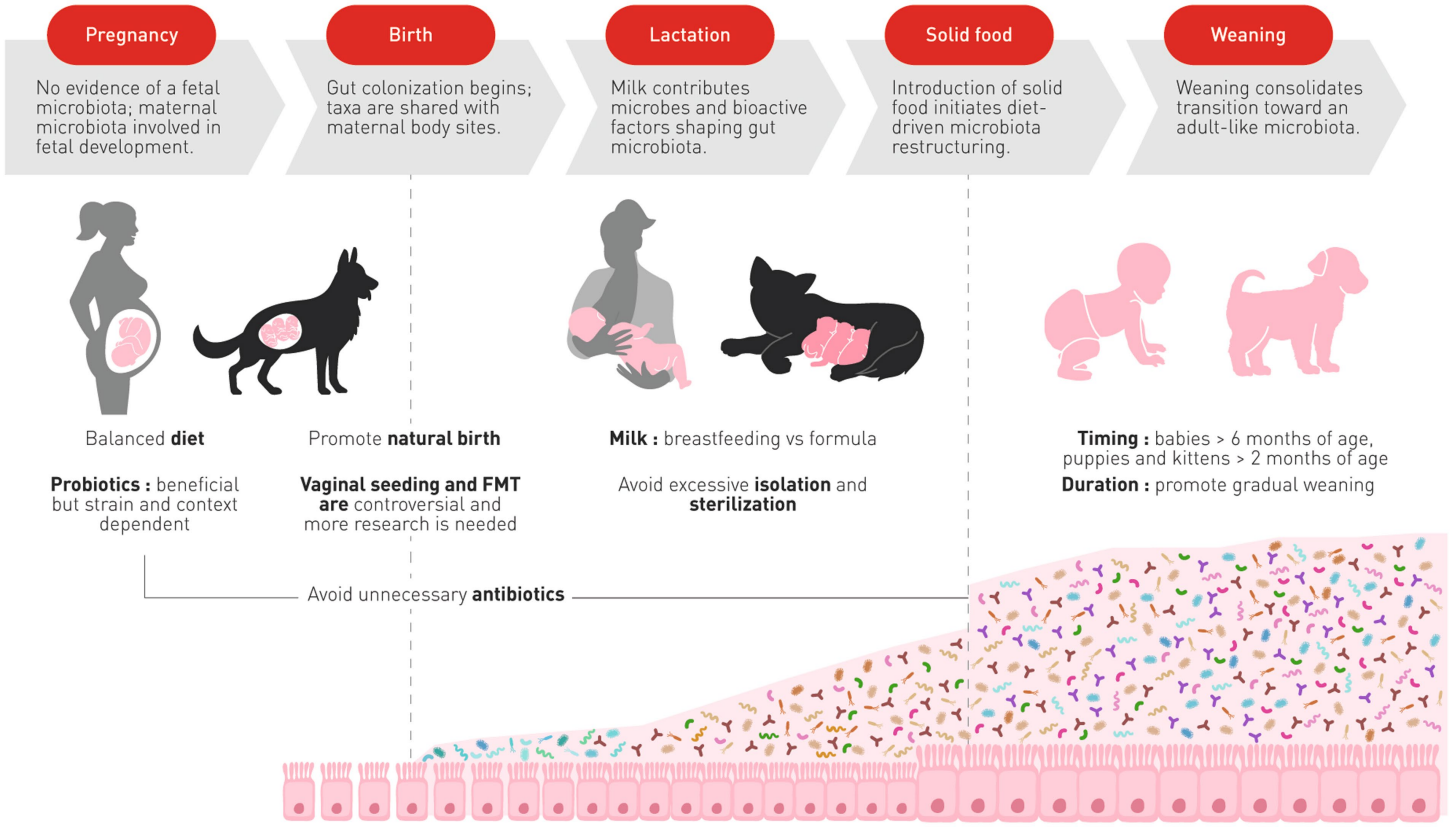
Perinatal shaping of the gut microbiota in companion animals and humans. This schematic summarizes the main stages of early-life microbial assembly from pregnancy to weaning and integrates current evidence-based concepts and practical considerations. First, key colonization events are illustrated along the perinatal timeline. Birth marks the onset of gut colonization through exposure to maternal microbiota and environmental sources. During lactation, colostrum and milk shape early microbial succession, while the introduction of solid food and weaning consolidate the transition toward a more stable, adult-like microbiota. Second, the figure highlights recommendations at each stage according to major factors of variation (i.e., maternal and neonatal diet, use of interventions such as antibiotics, probiotics and seeding techniques, birth mode, contact with sources of bacteria, timing and duration of weaning). Finally, the lower gradient illustrates the overall concept of holobiont shaping over time. Gut colonization begins at birth, and initial aerobic bacterial populations are substituted by anaerobic ones during lactation, while a gradual maturation of the gut epithelium occurs. The introduction of solid food starts the process of weaning, accompanied by a progressive increase in microbial diversity and functional complexity. This framework emphasizes how perinatal management acts not on isolated microbes, but on the entire holobiont and future health of the host.

Despite the growing interest, much remains to be understood to optimize perinatal microbiota management in dogs and cats. While research in humans provides valuable conceptual frameworks and hypotheses, further studies are needed to measure the long-term effects of the maternal and neonatal microbiota on canine and feline health. Perinatal microbiota dynamics and current evidence-based considerations in humans and companion animals are summarized in Figure 1, whereas Table 1 summarizes key determinants of perinatal microbiota development in humans, dogs, and cats. This is modulated by a combination of interconnected factors, including maternal microbiota, delivery mode, early-life nutrition, and postnatal environmental exposures. These factors act in a time-dependent manner during critical developmental windows, influencing microbial colonization patterns. From a clinical standpoint, optimizing maternal health and nutrition, ensuring timely and adequate colostrum intake, favoring maternal-neonatal contact, and carefully managing interventions such as cesarean sections, antibiotic and probiotic use, or artificial rearing may represent key strategies to support optimal microbiota development and improve neonatal outcomes. When conducting research on the perinatal microbiota, it is important to recognize that some biological sites represent zero- to low-biomass environments, making results highly susceptible to contamination during sample collection and processing (22, 23). Therefore, the inclusion of appropriate negative controls and the use of complementary methodological approaches are essential to minimize bias and avoid erroneous conclusions. Future research in companion animals should prioritize several key areas and developmental windows. First, in line with the DOHaD framework, species-specific investigations are needed to clarify the contribution of maternal gut and vaginal microbiota before and throughout gestation to offspring health in dogs and cats. This research should include longitudinal profiling of the maternal vaginal and gut microbiota in dams, coupled with long-term correlations to health outcomes, to link early-life microbial shifts with mechanistic signals relevant for future health in puppies and kittens. Furthermore, potential paternal contribution warrants investigation, as transgenerational effects of the paternal gut microbiota on offspring reproductive potential and somatic growth have been reported in mouse models (120). This may represent an area worth exploring in companion animals due to the importance of early growth and future ability to reproduce in breeding animals.

**Table 1:** Comparative overview of key determinants of perinatal microbiota development in humans, dogs, and cats.

Feature	Humans	Dogs	Cats
Evidence for in utero colonization	Not supported (22)	Not supported, colonization begins at birth (23)	Not supported (23)
Timing of colonization	Colonization begins at birth, dynamic changes in first months (74, 121)	Colonization begins at birth, dynamic changes in first weeks (100, 102, 103)	Colonization likely begins at birth, no specific studies
Main sources of initial microbiota at birth	Distributed across multiple maternal sites: maternal gut (22.1%), vagina (16.3%), skin, nasopharynx, and oral cavity (67, 74)	Predominantly from the dam’s vagina (71.6% match) (66)	Not investigated
Predominant bacterial groups in early neonatal gut	Initially dominated by facultative anaerobes like, which are progressively replaced by strictly anaerobic bacteria. Dominant *Bifidobacterium*, *Bacteroides*, *Escherichia* (77)	At birth, *Psychrobacter*, *Staphylococcus*, *Enterococcus*, *Escherichia*; early increases in *Peptostreptococcaceae*, *Pasteurellaceae*, and *Clostridiaceae* by 8 h. Evolution toward obligate anaerobes with consistently low levels of *Bifidobacterium* (66, 102)	Not investigated
Influence of delivery mode	C-section associated with reduced maternal transmission and delayed colonization; *Enterococcus*, *Streptococcus*; *Staphylococcus* (72, 76, 77)	90% bacterial growth in meconium of vaginally delivered puppies vs. 37% in CS-born puppies. CS-born puppies microbiota composition resembles the maternal skin and mouth (11, 24)	Not investigated
Role of colostrum and maternal milk	Provides immunoglobulins (IgA), microbes, and bioactive compounds shaping microbiota; dominance of beneficial bacteria (*Bifidobacterium*, *Lactobacillus*) in breastfed infants (93–95)	Colostrum is essential for passive immunity (limited transplacental Ig transfer); contributes to microbes and immune factors. Colostrum is dominated by *Psychrobacter*, whereas milk is dominated by *Staphylococcus*, abundance of *Bifidobacterium* remains consistently low (106, 118, 122)	Essential for passive immunity (limited transplacental Ig transfer); contributes microbes and immune factors (122)
Impact of early milk replacers	Associated with altered microbiota and enrichment in potentially pathogenic taxa (*Clostridioides*, *Enterobacteriaceae*) and antimicrobial resistance genes (93, 95)	Not investigated	Milk replacers affect gut microbiota composition in kittens (increase in *Lachnospiraceae*, *Enterococcus*, *Rothia* and *Ligilactobacillus*) (108)
Level of available evidence	Extensive, including longitudinal human cohorts	Emerging, increasing number of studies	Scarce; major research gaps

The table summarizes current evidence on the timing and sources of initial microbial colonization, early gut microbiota composition, and the effects of delivery mode, colostrum, maternal milk, and milk replacers on neonatal gut microbiota. Major knowledge gaps in companion animals, particularly in cats, are highlighted.

Second, well-designed, standardized, longitudinal clinical trials including next-generation sequencing methods should investigate the impact of maternal dietary interventions, including pre- and probiotic supplementation, on colostrum and milk microbiota and their effects on neonatal gut microbiota and health. Third, systematic molecular profiling of the kitten gut microbiota from birth to weaning remains largely unexplored and represents an important gap in current knowledge.

Finally, clinical trials are essential to evaluate the efficacy and safety of direct probiotic supplementation in puppies and kittens. Overall, research on perinatal microbiota holds the potential to significantly improve clinical veterinary practice. With reproductive microbiota now attracting growing attention and molecular tools for microbiome characterization becoming more widely available, establishment of longitudinal birth cohorts and collaborative multicentric studies are essential to fully the potential of this field.
