# Correction: The perinatal microbiota in dogs and cats: a narrative review from human research to veterinary practice

**DOI:** 10.3389/fvets.2026.1866749

**Published:** 2026-06-10

**Authors:** Penelope Banchi, Hanna Mila, Marta Selma-Royo, Smadar Tal, Franck Péron, Virginie Gaillard

**Affiliations:** 1 Faculty of Veterinary Medicine, Ghent University, Merelbeke, Belgium; 2 NeoCare, Université de Toulouse, ENVT, Toulouse, France; 3 Institute of Agrochemistry and Food Technology-Spanish National Research Council (IATA-CSIC), Paterna, Spain; 4 Koret School of Veterinary Medicine, The Hebrew University Veterinary Teaching Hospital, Hebrew University of Jerusalem, Rehovot, Israel; 5 Tel-Hai Academic College, Upper Galilee, Israel; 6 Royal Canin Research Center, Aimargues, France

**Keywords:** bacteria, canine, feline, fetal, microbiome, microbiota, neonatal, perinatal period

There was a mistake in Figure 1 as published. The timing of weaning for puppies and kittens was displayed as “>3 old.” The correct statement is “>2 months of age.” The corrected Figure 1 and its caption appear below.

**Figure 1:**
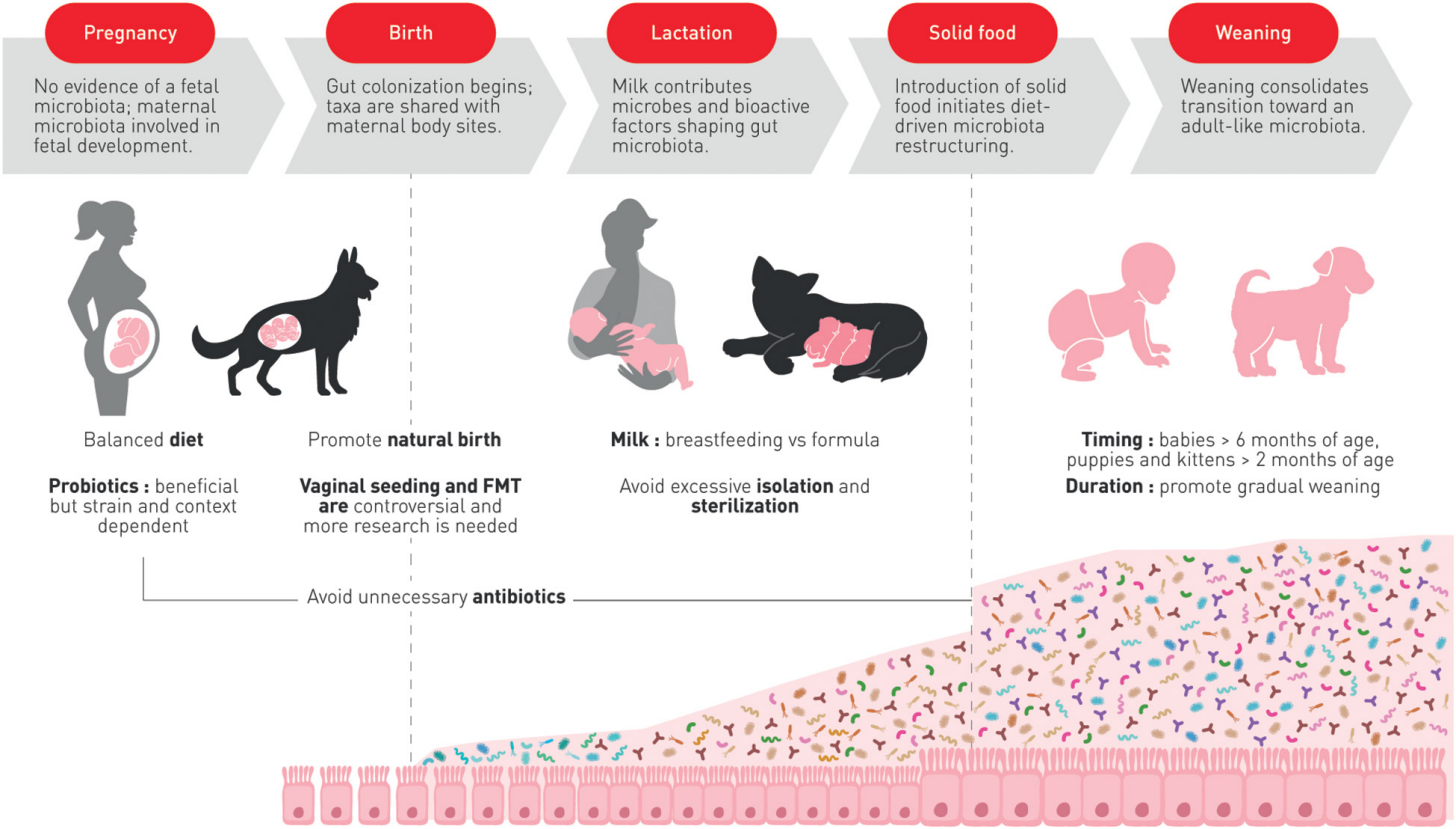
Perinatal shaping of the gut microbiota in companion animals and humans. This schematic summarizes the main stages of early-life microbial assembly from pregnancy to weaning and integrates current evidence-based concepts and practical considerations. First, key colonization events are illustrated along the perinatal timeline. Birth marks the onset of gut colonization through exposure to maternal microbiota and environmental sources. During lactation, colostrum and milk shape early microbial succession, while the introduction of solid food and weaning consolidate the transition toward a more stable, adult-like microbiota. Second, the figure highlights recommendations at each stage according to major factors of variation (i.e., maternal and neonatal diet, use of interventions such as antibiotics, probiotics and seeding techniques, birth mode, contact with sources of bacteria, timing and duration of weaning). Finally, the lower gradient illustrates the overall concept of holobiont shaping over time. Gut colonization begins at birth, and initial aerobic bacterial populations are substituted by anaerobic ones during lactation, while a gradual maturation of the gut epithelium occurs. The introduction of solid food starts the process of weaning, accompanied by a progressive increase in microbial diversity and functional complexity. This framework emphasizes how perinatal management acts not on isolated microbes, but on the entire holobiont and future health of the host.

The original version of this article has been updated.

